# Successful treatment of 2 patients with brain metastases from non-small cell lung cancer with epidermal growth factor receptor mutation receiving dacomitinib: A case report

**DOI:** 10.1097/MD.0000000000026680

**Published:** 2021-07-30

**Authors:** Songchen Zhao, Xiaofeng Cong, Ziling Liu

**Affiliations:** Deparment of Oncology, First Affiliated Hospital, Jilin University, Changchun, Jilin, PR China.

**Keywords:** brain metastases, case report, dacomitinib, non-small cell lung cancer

## Abstract

**Rationale:**

Approximately 20% of patients with non-small cell lung cancer (NSCLC) are diagnosed with brain metastasis, which is related to poor survival outcomes. The ability of tyrosine kinase inhibitor drugs to penetrate the blood–brain barrier makes them a potential option for intracranial metastases. Dacomitinib, an irreversible second-generation pan-HER tyrosine kinase inhibitor, has become a standard therapy for patients with epidermal growth factor receptor mutations. However, its efficacy in patients with brain metastases (BMs) is not yet established. Here, we present 2 patients with epidermal growth factor receptor-mutant NSCLC with brain metastasis. After initiation of dacomitinib as first-line treatment, a significant clinical response was achieved, and a long-lasting complete remission was achieved in 1 patient up to this date.

**Patient concern:**

Case 1 was a 47-year-old man who was admittedtothe hospital because of recurrent cough and expectoration for >1 year. Chest computed tomography scans revealed a high-density shadow in the left upper lobe. Cranial magnetic resonance imaging indicated an abnormal nodular enhancement in the right cerebellar hemisphere. Case 2 was a 55-year-old man with a chief complaint of intermittent cough and expectoration for >1 month. Chest computed tomography revealed a high-density mass in the left superior lobe. Magnetic resonance imaging of the central nervous system revealed 2 abnormal nodular enhancements in the left frontal lobe.

**Diagnosis:**

Both patients were diagnosed with lung adenocarcinoma by bronchoscopy and lymph node biopsy.

**Interventions:**

Both patients received dacomitinib 30 mg once daily as first-line therapy for 8 and 11 months, respectively until disease progression.

**Outcome:**

After treatment with dacomitinib, both patients achieved complete response in BMs. Progression-free survival was 11 and 8 months, respectively.

**Lessons:**

Dacomitinib strongly controlled BMs in patients with advanced NSCLC, and the adverse reactions were tolerable. Dacomitinib may be considered a new treatment option for these patients. Further prospective studies are recommended to confirm this conclusion.

## 1 Introduction

Lung cancer remains to be the leading cause of cancer-related deaths worldwide.^[1]^ Brain metastases occur in up to 50% of patients during the course of the disease.^[2]^ It has always been a conundrum in the treatment of non-small cell lung cancer (NSCLC) due to its effect on drug penetration to the blood–brain barrier (BBB) into the central nervous system. Previous studies have shown that brain metastasis is a negative prognostic factor for lung cancer and is associated with poor survival outcomes.^[3]^ Surgical resection, stereotactic radio surgery, and whole-brain radiation therapy have been the traditional therapies for NSCLC patients with brain metastases (BMs). As we learn more about the genetic and molecular mechanisms that underlie the development of the disease, tyrosine kinase inhibitor drugs have become a new possible treatment option for NSCLC patients with BMs. Dacomitinib, a second-generation, irreversible, and highly selective pan-HER tyrosine kinase inhibitor orally-administered drug, has been shown to penetrate the BBB in preclinical trials.^[4]^ However, the efficacy in patients with BMs remains unclear because these patients were excluded from related clinical trials. Here, we report 2 epidermal growth factor receptor (EGFR)-mutant NSCLC patients with brain metastases who responded well to dacomitinib. Consent was obtained from 2 patients for publication purposes.

## 2 Case presentation

### 2.1 Case 1

**Figure 1:**
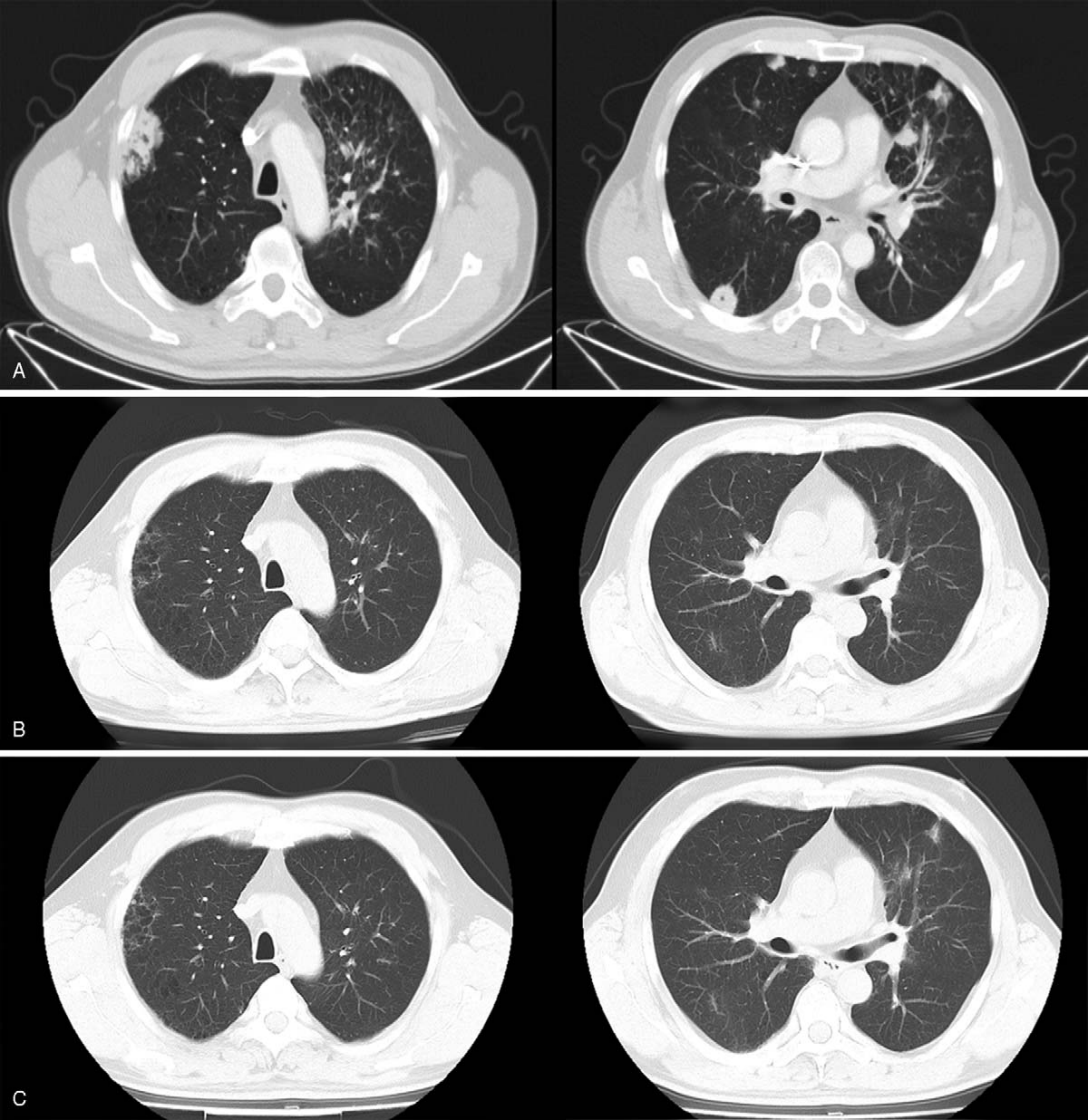
Chest CT imaging results for the case 1 during treatment with dacomitinib. (A) Chest CT scans revealed a high-density shadow in the left upper lobe, accompanied by multiple nodules in both lungs at diagnose (December 26, 2019). (B) Two months after treatment, the mass in the upper lobe of the left lung was significantly reduced in size, and the bilateral pulmonary nodules almost disappeared. (C) At the time of disease progresses (November 29, 2020), the volume and number of multiple pulmonary nodules in both lungs were increased, the mass in the upper lobe of the left lung remained stable. CT = computed tomography.

**Figure 2:**
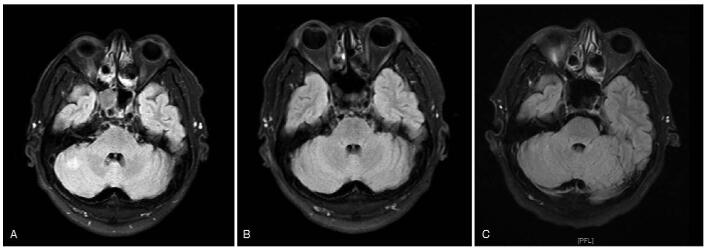
Head MRI imaging results for the case 1 during treatment with dacomitinib. (A) Brain MRI indicated the metastases in the right cerebellar hemisphere at diagnose (December 26, 2019). (B) The brain metastases were reduced prominently after 2 months of dacomitinib treatment, which was evaluated as a CR. (C) Head MRI showed no recurrence of BMs at the time of disease progresses (November 29, 2020). CR = complete response, MRI = magnetic resonance imaging

A 47-year-old man was admitted to the hospital in December 2019 due to recurrent cough and expectoration for >1 year. He had a history of smoking for over 30 years. Chest computed tomography (CT) revealed a high-density shadow in the left upper lobe, which was pathologically confirmed as lung adenocarcinoma by transbronchial biopsy. Multiple nodular high-density shadows were observed in each lobe of both lungs; the sizes range between 0.4 and 4.0 cm. Some were vacuoles, which were augmented by enhanced scanning (Fig. 1A). Cranial magnetic resonance imaging (MRI) indicated an abnormal nodular enhancement in the right cerebellar hemisphere and multiple enlarged left cervical lymph nodes (Fig. 2A). Abdominal CT and bone scan did not reveal any other metastases. Based on the above examination results, the tumor was classified as stage IV-B (cT4NxM1c) lung adenocarcinoma. Gene sequencing of the tumor confirmed the exon 19 deletion of EGFR. The patient was given 30 mg of dacomitinib per day as a first-line therapy since January 2020. Two months after the treatment, brain metastasis was undetectable on MRI (Fig. 2B). This was considered a complete response (CR). Chest CT also illustrated that the lung lesions had significantly decreased in size, and the overall efficacy was classified as partial remission (PR) according to the Response Evaluation Criteria in Solid Tumors version 1.1 (Fig. 1B). During the treatment with dacomitinib, the patient developed slight rash, diarrhea, and paronychia, which were tolerable and did not result in termination of treatment or dose reduction. Regular monitoring every 2 months showed that the disease did not progress until the end of November 2020.

On November 29, 2020, after 11 months treatment of dacomitinib therapy, the repeat lung CT scan showed an increase in the volume and number of nodules in both lungs, suggesting the progression of lung cancer (Fig. 1C). Cranial MRI showed no recurrence of BMs (Fig. 2C), and further examination revealed no other distant metastases. Genetic testing was performed again, and the results were consistent with the initial diagnosis but without the T790M mutation. The progression-free survival (PFS) of the patient on first-line dacomitinib was 11 months. However, the patient discontinued dacomitinib therapy after disease progression. Although no T790M mutation was found in the second genetic test, the patient insisted on taking osimertinib. Due to the patient's personal reasons, an imaging review was not performed until the submission of the manuscript.

### 2.2 Case 2

**Figure 3:**
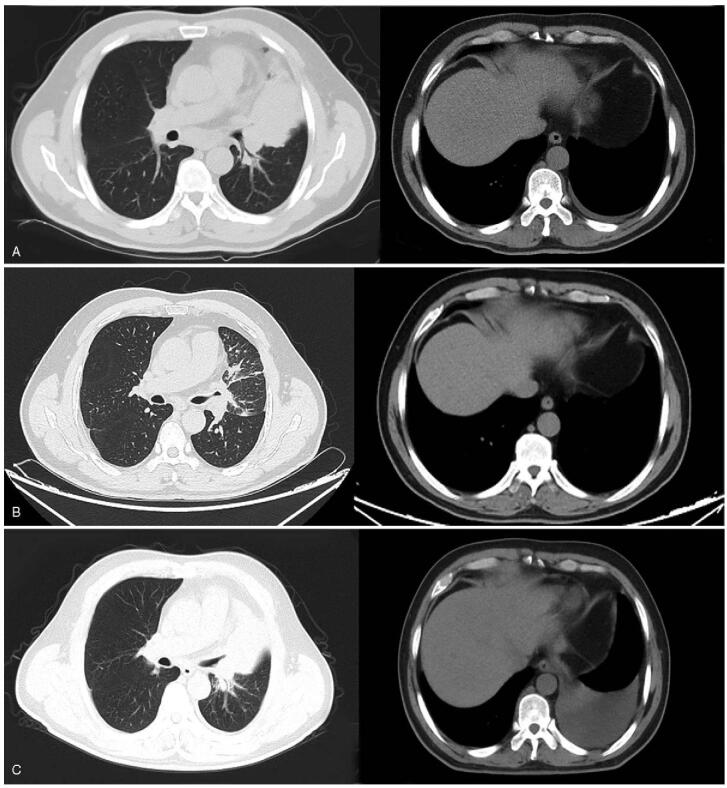
Chest CT imaging results for the case 2 during treatment with dacomitinib. (A) Chest CT showed a high density mass in the left superior lobe accompanied by enlarged mediastinum, left hilum lymph nodes; a mild left pleural effusion was also detected at diagnose (April 20, 2020). (B) Three months after treatment, chest CT showed a remarkable reduction in the lung lesions, and pleural effusion was completely disappeared, the efficacy was evaluated as a PR (July 25, 2020). (C) A repeat CT scan indicated progression of lung lesions, the left pleural effusion occurred again, and the mass in the left upper lobe of the lung was enlarged (December 30, 2020). CT = computed tomography, PR = partial remission.

**Figure 4:**
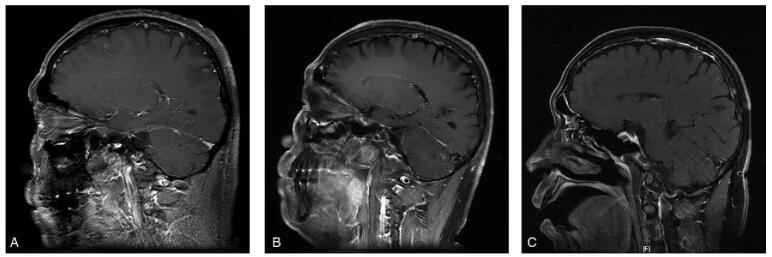
Head MRI imaging results for the case 2 during treatment with dacomitinib. (A) Brain MRI revealed 2 abnormal nodular enhancements in the left frontal lobe at diagnose (April 20, 2020). (B) Three months after treatment, brain MRI showed disappearance of the intracranial metastases, which was judged as a CR. (C) MRI indicated 2 nodules of abnormal enhancement in the right frontal lobe at the time of disease progression (January 7, 2021). CR = complete response, MRI = magnetic resonance imaging.

A 55-year-old man with no history of smoking was admitted to our hospital in April 2020 due to intermittent cough and expectoration for >1 month. Chest CT showed a high-density mass in the left superior lobe accompanied by an enlarged mediastinum, left hilum, and supraclavicular lymph nodes with mild left pleural effusion (Fig. 3A). Percutaneous lymph node biopsy was performed on the larger node, and the pathology confirmed lung adenocarcinoma. MRI of the central nervous system revealed 2 abnormal nodular enhancements in the left frontal lobe (Fig. 4A). Based on the above results, the tumor was classified as stage IV-B (cT4N3M1c) lung adenocarcinoma. Genetic testing showed EGFR exon 21 L858R mutation. The patient began taking dacomitinib (30 mg/d) on May 1, 2020. Three months later, cranial MRI showed disappearance of the intracranial metastases, which was considered CR (Fig. 4B). Chest CT revealed a remarkable reduction in the lung lesions, and the pleural effusion completely disappeared. The overall efficacy was classified as a PR (Fig. 3B). The patient developed mild rash, paronychitis, and oral mucositis during the treatment, which did not result in dose reduction of dacomitinib. Regular imaging was performed every 2 months. On December 30, 2020, a repeat CT scan indicated progression of lung lesions, recurrence of the left pleural effusion, and an enlarged mass in the left upper lobe (Fig. 3C). MRI indicated progression of head lesions on January 7, 2021 (Fig. 4C). This patient had a PFS of 8 months on dacomitinib. Genetic testing was performed again and the T790M mutation was confirmed. The oral chemotherapeutic drug was replaced with osimertinib from January 8, 2021 until now.

## 3 Discussion

Approximately 20% of patients with NSCLC have BM at the time of diagnosis.^[5]^ Due to the BBB, intracranial metastases have always been an obstacle in the treatment of NSCLC, and is associated with poor prognosis. The permeability of the BBB remains a challenge for small-molecular-targeted anti-tumor drugs. The efficacy of first-generation EGFR inhibitors for intracranial metastases in advanced NSCLC is not significant. One of the blocking effects of BBB on intracranial lesions is through the P-glycoprotein (ABCB1) and the breast cancer resistance protein (ABCG2)-mediated drug effusion. Gefitinib and erlotinib are substrates of ABCB1 and ABCG2 transporters, hence their distribution to the lesions in the brain is limited.^[6,7]^

Fan et al^[8]^ found that dacomitinib was an inhibitor of ABCB1 and ABCG2, which restrained drug efflux in cell lines with overexpressed transporters. After oral administration of a single dose of [14C] dacomitinib in male Long-Evans rats, the radioactivity derived from dacomitinib can be detected in the central nervous system and cerebrospinal fluid within 2 to 48 hours, confirming its ability to cross the BBB (data not published/investigator brochure provided by Pfizer). A preclinical study showed that dacomitinib prolonged the survival of mice with patient-derived xenograft models of glioblastoma. Additionally, immunofluorescence staining and contrast-enhanced MRI confirmed that the intracranial tumor load of mice treated with dacomitinib was significantly reduced.^[9]^ Chi et al^[10]^ confirmed the permeability of the BBB by measuring the level of endadatinib in tumors after treatment. These data indicate the ability of dacomitinib to penetrate the BBB effectively, both in rats and humans, and its therapeutic effect on intracranial lesions. These findings provide evidence on the response of intracranial metastases to dacomitinib. The ARCHER1050 study showed that compared with gefitinib, dacomitinib was a better choice for patients with EGFR-mutant NSCLC, which significantly improved patients’ PFS.^[11]^ However, the ARCHER1050 study excluded patients with brain metastases. Therefore, the effect of dacomitinib on the central nervous system is unclear.

Here, we present 2 clinical cases that responded significantly to dacomitinib and achieved CR of the intracranial lesion. The 2 patients had PFS of 11 and 8 months, respectively, and none of the brain metastases reappeared at the time of the first disease progression. Patients did not receive any anti-tumor therapy before dacomitinib. Therefore, the effect on intracranial lesions was not due to the destruction of the BBB secondary to chemotherapy or radiotherapy. Our results further validate that dacomitinib can penetrate the BBB and provide a feasible new option for patients with brain metastases from NSCLC.

## Author contributions

**Conceptualization:** Xiaofeng Cong.

**Data curation:** Songchen Zhao.

**Investigation:** Songchen Zhao.

**Software:** Songchen Zhao.

**Supervision:** Xiaofeng Cong.

**Visualization:** Songchen Zhao.

**Writing – original draft:** Songchen Zhao.

**Writing – review & editing:** Ziling Liu.
